# Is Mandibular Cortical Bone and Trabecular Microarchitecture Altered by Masseter Botox Treatment? A Quantitative Perspective

**DOI:** 10.3390/diagnostics15172201

**Published:** 2025-08-29

**Authors:** Ibrahim Burak Yuksel, Fatma Altiparmak, Serkan Bahrilli, Fatma Ucan Yarkac, Dilek Ozkan Sen, Kaan Orhan

**Affiliations:** 1 Department of Dentomaxillofacial Radiology, Faculty of Dentistry, Necmettin Erbakan University, Konya 42090, Turkey; serkanbahrilli@gmail.com; 2 Department of Periodontology, Faculty of Dentistry, Necmettin Erbakan University, Konya 42090, Turkey; fatma_ucan413@hotmail.com (F.U.Y.);; 3 Department of Dentomaxillofacial Radiology, Faculty of Dentistry, Ankara University, Ankara 06500, Turkey; call53@yahoo.com

**Keywords:** bone remodeling, botulinum toxin type A, bruxism, fractal analysis

## Abstract

**Background/Objectives:** Bruxism is a prevalent parafunctional activity that induces masticatory muscle hypertrophy and physiological mandibular bone alterations. While botulinum toxin type A (BTX-A) injections effectively reduce muscle hyperactivity, objective radiological assessment of their skeletal effects remains limited. This study aimed to quantitatively evaluate the impact of BTX-A on mandibular trabecular bone structure by assessing potential reductions in fractal dimension (FD) in bruxism patients compared to controls. **Methods:** This retrospective matched-pair study included 42 bruxism patients (39 females, 3 males) receiving 50 U of BTX-A in masseter muscles and 32 age-matched non-bruxism controls (30 females, 2 males). Pre- and 6-month post-treatment panoramic radiographs were analyzed. FD values were calculated from bilateral condylar neck, ramus, and angulus regions. **Results:** Statistically significant reductions in FD values were observed in the angulus regions post-treatment (Right: 1.3856 to 1.2995; Left: 1.3718 to 1.2529, *p* < 0.001). Postoperative FD values in the BTX-A group showed no significant differences when compared to the control group (*p* > 0.05). **Conclusions:** BTX-A administration leads to measurable, region-specific reductions in mandibular trabecular bone fractal dimension, particularly in the angulus. The postoperative similarity to the control group suggests a potential normalization of bone structure following treatment. These findings highlight the clinical utility of fractal analysis for monitoring osseous adaptations in neuromuscular interventions.

## 1. Introduction

Bruxism, defined by the American Academy of Orofacial Pain as a parafunctional activity of the masticatory muscles, involves repetitive actions like clenching, grinding, bracing, or thrusting of the jaw. This activity can manifest rhythmically or irregularly during sleep (nocturnal) or wakefulness (diurnal) [1]. Studies estimate that approximately 80–95% of the world’s population experiences sleep bruxism, being more common in individuals aged 15–40, and is a clinically important phenomenon as it is commonly encountered in the adult population [2,3]. Symptoms associated with bruxism encompass tooth wear, dental hypersensitivity, fracture of restorations, muscle pain, and temporomandibular joint (TMJ) discomfort, underscoring its complex and multifaceted nature [4,5]. The etiology is generally regarded as a combination of biological, external, and psychological factors, including alcohol, tobacco, certain medications, oral habits, TMJ disorders, malocclusion, high anxiety levels, psychiatric disorders, and stress [6]. One of the most prevalent consequences of bruxism is masticatory muscle hypertrophy, particularly affecting the masseter muscle. Over time, this hypertrophy may induce physiological alterations in the trabecular structure of the bone [7,8]. Both surgical and non-surgical interventions are employed for the management of masseter hypertrophy; however, surgical procedures carry risks such as asymmetry, condylar fracture, and damage to the inferior alveolar nerve [9]. Consequently, botulinum toxin type A (BTX-A) injections have emerged as a safer, reversible, non-surgical alternative. BTX-A functions by inhibiting the release of acetylcholine at neuromuscular junctions, resulting in muscle paralysis and atrophy [10]. This mechanism diminishes muscle contractions associated with bruxism and influences bone remodeling processes. Empirical studies demonstrate that BTX-A injections markedly reduce muscle thickness, cross-sectional area, and condylar trabecular bone density following treatment. Furthermore, research indicates that hyperactivity of the masseter attributable to bruxism increases bone density at sites of muscle attachment, and that BTX-A injections mitigate this density by inducing modifications in the bone structure within these regions [9,11].

However, to objectively quantify these subtle microarchitectural changes, advances in digital image processing techniques, such as fractal analysis (FA), have facilitated the detection of details in radiographs that may be overlooked in two-dimensional imaging, while still providing valuable information regarding the three-dimensional structure of bone [12]. FA quantifies the complexity of bone architecture as a numerical value and is frequently employed for assessing bone density [13,14]. FA is extensively utilized for evaluating the internal trabecular structure of the mandible due to its non-invasive methodology, insensitivity to variables such as projection geometry and radiation dose, and its ability to furnish objective data. Furthermore, FA serves as an effective technique for monitoring changes in bone density following treatment [15]. As the complexity of the analyzed structure increases, so does the fractal dimension (FD) value; a higher FD signifies denser bone with smaller voids, whereas a lower FD indicates more porous bone with larger voids, often associated with reduced bone density or altered trabecular architecture [15,16].

Despite growing evidence of BTX-A’s influence on masticatory muscles, quantitative radiological assessments specifically exploring its effects on human mandibular bone microarchitecture, especially with detailed analyses like FD, remain limited and sometimes inconsistent in the literature. This lack of clear and consistent data, often stemming from the retrospective nature of existing studies and a lack of control for potential confounding variables, underscores the necessity for further robust investigations into the skeletal implications of BTX-A treatment in bruxism patients [17].

The primary objective of this study is to investigate alterations in mandibular bone trabecular structure, specifically within the regions of the angle, ramus, and condylar neck, through the application of fractal dimension calculations. This investigation will focus on: (1) comparing pre-treatment and 6-month post-treatment FD values in bruxism patients receiving BTX-A injections; (2) comparing postoperative FD values between BTX-A treated bruxism patients and a control group without bruxism.

Our null hypothesis stated there would be no statistically significant differences in FD values across the selected regions of interest within the mandible (condylar neck, ramus, and angulus). This applies both between pre- and post-treatment measurements in bruxism patients and between BTX-A-treated bruxism patients and non-bruxism controls at the conclusion of the six-month follow-up.

## 2. Materials and Methods

### 2.1. Study Design and Ethical Considerations

This retrospective observational study was conducted in accordance with the ethical principles outlined in the Declaration of Helsinki (2013 revision) and was approved by the Clinical Research Ethics Committee of Necmettin Erbakan University Faculty of Dentistry (Decision No: 2025/572; Date: 27 March 2025). Following ethical approval, radiographic records archived at the Department of Oral and Maxillofacial Radiology were retrospectively reviewed.

Panoramic radiographs (PRs) of individuals who presented for routine dental examinations between 1 January 2024, and 10 March 2025, were retrospectively evaluated. Informed consent for both panoramic radiographic examination and BTX-A application is routinely obtained from all patients at our faculty as part of their initial visit and treatment plan. For this retrospective study, all radiographic data was collected from the institution’s archive. The 6-month follow-up PRs were taken as a standard component of our clinic’s post-treatment monitoring protocol to evaluate the long-term effects of BTX-A, in line with the initial consent provided. The control group consisted of a random selection of PRs from the archive belonging to patients who presented for routine dental examinations and had no history of bruxism or BTX-A treatment.

To evaluate the adequacy of the available sample, a post hoc power analysis was conducted using G*Power 3.1 based on the primary within-group comparison (paired samples *t*-test) of fractal dimension values in the angulus region of the bruxism group. The observed effect size for the right angulus was large (Cohen’s dz = 1.15), and the achieved sample size (*n* = 42) provided a statistical power greater than 99% at α = 0.05. For the between-group comparison of postoperative values in the same region (independent samples *t*-test, BTX-A vs. control), the observed effect size was small-to-moderate (Cohen’s d = 0.32), with the available sample sizes (*n* = 42 vs. *n* = 32) corresponding to approximately 60% power at α = 0.05.

### 2.2. Patient Selection

The study population was stratified into two distinct cohorts: a bruxism group and a control group:

Bruxism Group: This group consisted of 42 individuals (39 females and 3 males) who received BTX-A injections into the masseter muscle specifically for myofascial pain of bruxism origin. Bruxism diagnosis for these participants was established through a comprehensive assessment based on criteria outlined in the International Consensus on the Assessment of Bruxism report.

This assessment involved both anamnesis and clinical examination. During anamnesis, patients were queried about clenching or grinding habits (day or night), partner-reported grinding sounds during sleep, and any jaw pain or fatigue upon waking or during the day. Clinical examination included evaluation of dental attrition on functional and non-functional cusps, presence of prominent linea alba on the cheek mucosa, tooth indentations on the tongue border, hypertrophy of the temporal and masseter muscles, and pain or tenderness on palpation of the masticatory muscles.

A diagnosis of bruxism was confirmed if at least one anamnesis finding was present, combined with either dental attrition and/or masticatory muscle tenderness on clinical examination [1].

For these participants, both pre-treatment and 6-month post-treatment PRs were included for analysis.

Control Group: This group consisted of 32 individuals (30 females and 2 males) who exhibited no clinical signs or symptoms indicative of bruxism, a status confirmed through comprehensive clinical and radiographic evaluations. Specifically, control subjects reported no clenching or grinding habits, and exhibited no signs of dental attrition, linea alba, or masticatory muscle tenderness/hypertrophy on examination. This cohort was selected from patients undergoing routine dental examinations and had no documented history of bruxism diagnosis or therapeutic intervention. To ensure a valid comparative analysis, the control group was meticulously matched to the bruxism group based on age.

Participants included in the study were aged between 18 and 65 years, possessed PRs of sufficient diagnostic quality, and had no history of maxillofacial trauma, craniofacial deformities, or orthognathic surgery. To ensure a valid comparative analysis, the control group was meticulously matched to the bruxism group based on age. Furthermore, strict exclusion criteria were applied to both cohorts to minimize the influence of confounding factors, such as systemic diseases and pharmacological agents that could affect bone metabolism.

Conversely, patients were excluded from the study if their panoramic radiographs were of inadequate diagnostic quality, if they fell outside the 18 to 65 year age range, or if they presented with any of the following additional criteria:

- The presence of systemic diseases (e.g., osteoporosis, metabolic bone diseases) known to influence bone metabolism.
- A history of systemic glucocorticoid therapy or any other pharmacological intervention recognized to affect bone metabolism.
- Completely or partially edentulous areas that could impede the accurate assessment of mandibular bone density.
- The presence of intrabony lesions or bone fractures within the mandible.
- A planned regimen of extensive dental treatment during the study period that could potentially alter bone structure.

### 2.3. BTX-A Injection Procedure

In the bruxism group, phase I periodontal therapy was completed prior to the planning and administration of BTX-A injections into the masseter muscles by the Department of Periodontology. The primary goal of the BTX-A injections was to reduce muscular hypertrophy and alleviate related myofascial pain originating from bruxism. All patients were informed about the potential side effects of BTX-A injections and provided written informed consent before the procedure.

The BTX-A treatment consisted of 100 U of frozen dry BTX-A (Botox, Allergan, Inc., Irvine, CA, USA) diluted with 2 cc saline solution to obtain a 1.0 U/0.1 mL dose. A total of 50 U of BTX-A was injected, with 25 U administered into each masseter muscle, using 30-gauge injectors at 3 distinct points per muscle. All injection procedures were performed by the same experienced periodontist (D.O.S.).

### 2.4. Image Acquisition

All radiographs were obtained using a Planmeca ProOne^®^ panoramic imaging device (Planmeca Oy, Helsinki, Finland). The device was operated at 68 kVp, 7 mA, with a 10-s exposure period. The same experienced technician operated the device throughout the study to ensure consistency.

The device was operated exclusively by the same experienced technician throughout the study. Image optimization, including adjustments to brightness, contrast, and filtration, was carried out utilizing Romexis^®^ software (version 6.4.7; Planmeca, Helsinki, Finland). Raw, unprocessed image data were employed for subsequent analyses.

### 2.5. Image Processing and Fractal Analysis

**Figure 1:**
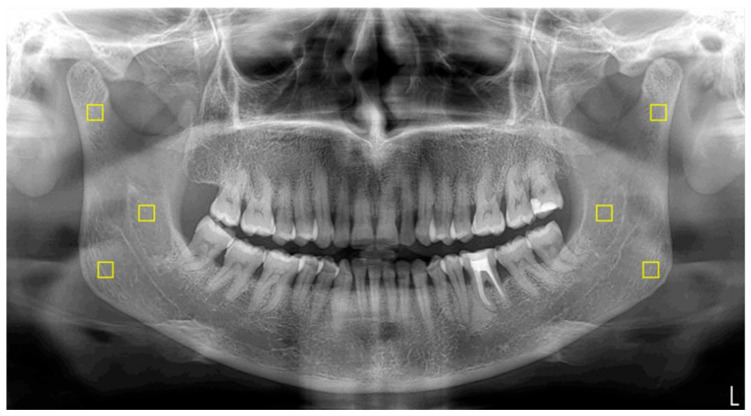
ROIs selected from the right and left condylar, mid-ramus, and angulus bone areas in a panoramic image. The ROIs are highlighted with yellow squares.

**Figure 2:**
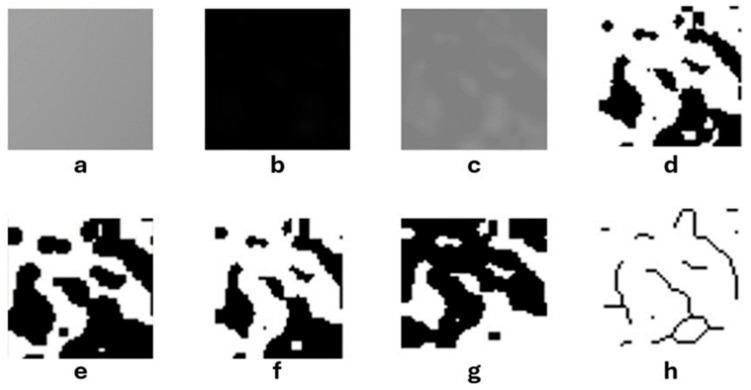
FA process steps: (**a**) cropped, duplicated and Gaussian blur filtered region of interest image; (**b**) extracting the blurred image from the duplicated ROI image; (**c**) adding 128 gray tones; (**d**) converting to black and white image; (**e**) erode process; (**f**) dilate process; (**g**) color inversion and (**h**) skeletonization.

**Figure 3:**
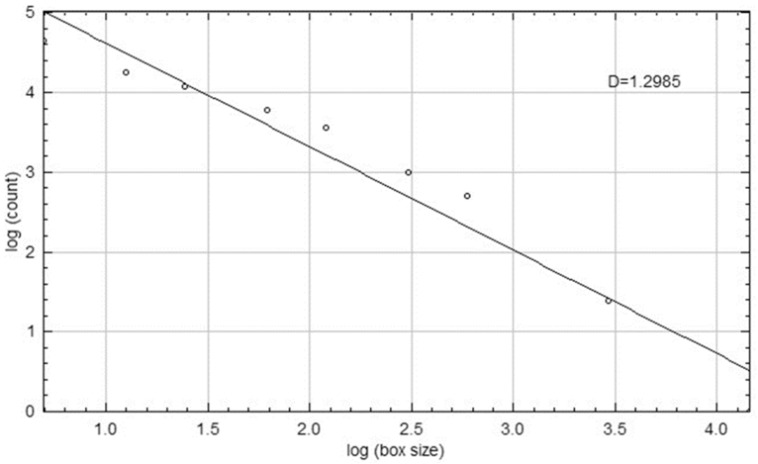
The FD calculated from the slope of the line that best fits the points obtained from the logarithmic scale graph of the values was expressed as the ‘D’ value.

The standardization of panoramic images was executed using PhotoScape X Pro 4.2.7. software (MOOII Tech, Seoul, Republic of Korea). All images were resized to 1024 × 499 pixels at a resolution of 600 dpi and saved in TIF format. FD values were determined utilizing ImageJ version 1.54 software, implementing the box-counting method introduced by White and Rudolph [18,19]. Regions of interest (ROIs) measuring 25 × 25 pixels were bilaterally selected from the mandibular angle, ramus, and condylar neck (Figure 1). Each ROI was subjected to digital preparation, including rotation, magnification, cropping, and duplication. A Gaussian blur filter was applied to reduce grayscale density variations and to emphasize trabecular structures (Figure 2). The resulting values were then plotted on a logarithmic scale to determine the slope of the line that best fit the points on the graph, which was defined as the FD (Figure 3).

To ensure measurement reliability, 20% of the images were randomly chosen and independently reanalyzed by two observers (I.B.Y. and S.B.), each performing two rounds of analysis. All assessments were conducted on a computer system equipped with Windows 11, 8 GB RAM, and an NVIDIA GeForce RTX 3050 graphics card.

### 2.6. Statistical Analysis

All statistical analyses were performed using IBM SPSS Statistics version 21.0 (IBM Co., Armonk, NY, USA). Descriptive statistics (mean ± standard deviation) were calculated for all variables. The normality of data distribution was assessed using the Kolmogorov–Smirnov test. The reliability of repeated FD measurements by the same observer was evaluated using the intraclass correlation coefficient (ICC). In the bruxism group, pre- and post-treatment FD values were compared using the paired samples *t*-test. Between-group comparisons of postoperative FD values (BTX-A group vs. non-bruxism controls) were conducted using the independent samples *t*-test. Additionally, sex-based comparisons of pre- and post-treatment FD values within the BTX-A group were analyzed using the Mann–Whitney U test. A *p*-value less than 0.05 was considered statistically significant.

## 3. Results

This study included 42 bruxism patients (39 females, 3 males) and 32 age-matched non-bruxism controls (30 females, 2 males). The mean age of the bruxism group was 35.73 years (18–65 years). Intraobserver reliability for repeated FD measurements, assessed on a randomly selected subset of regions of interest, was found to be satisfactory, with an intraclass correlation coefficient (ICC) of 0.841 (95% Confidence Interval: 0.782–0.892, *p* < 0.001).

### Fractal Dimension (FD) Analysis

**Figure 4:**
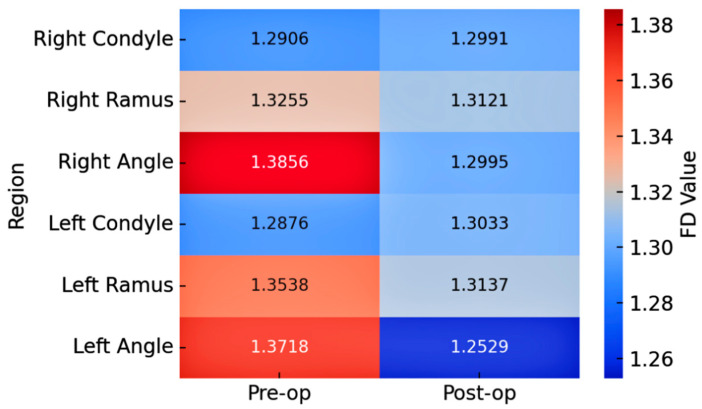
Heatmap illustrating the mean FD values of mandibular regions in bruxism patients before and after BTX-A treatment. Warmer colors represent higher FD values, while cooler colors indicate lower values. Notable reductions are observed in the right and left angulus regions post-treatment, consistent with the statistical analyses.

Table 1 presents the changes in FD values for the mandibular regions of bruxism patients following BTX-A treatment. A statistically significant reduction in FD values was observed exclusively in the angulus regions (*p* < 0.001 for both right and left sides). The mean FD value on the right side decreased from 1.3856 ± 0.0896 to 1.2995 ± 0.1225, while on the left side, it decreased from 1.3718 ± 0.0726 to 1.2529 ± 0.1024. This significant reduction in the angulus region is also visually supported by the heatmap in Figure 4, which shows a clear change from warmer to cooler colors, indicating a decrease in FD values. No statistically significant changes were found in the condylar neck and ramus regions (*p* > 0.05).

Table 2 presents a comparison of the postoperative fractal dimension values of the mandibular regions between bruxism patients treated with botox and control subjects without a bruxism diagnosis. No statistically significant differences were observed in the mean values of the right and left condyle, ramus, and angulus regions between the two groups (*p* > 0.05). These findings indicate that the trabecular bone structure exhibited similar fractal characteristics in both groups following BTX-A treatment in the bruxism group. This suggests that BTX-A treatment may help normalize or preserve the mandibular trabecular bone microarchitecture in bruxism patients, aligning it with that of individuals without the condition.

## 4. Discussion

One of the most noteworthy aspects and primary finding of our study is the quantitative evaluation of BTX-A’s impact on mandibular bone microarchitecture through FA of PRs. The rising prevalence of bruxism, often linked to modern lifestyle stressors, exerts detrimental effects on the stomatognathic system, manifesting as myofascial pain and masticatory muscle hypertrophy, particularly of the masseter [5,17]. Untreated, this muscular hyperactivity can lead to significant physiological alterations in mandibular bone structure. While various symptomatic treatments exist, BTX-A injection into the masseter muscle has emerged as a minimally invasive and effective alternative for reducing muscular hyperactivity by inhibiting acetylcholine release, thereby decreasing excessive mechanical load on adjacent bone structures. To quantitatively assess BTX-A’s impact on bone, FA offers a non-invasive and reproducible method to evaluate mandibular microstructural changes. FA quantifies trabecular bone complexity through FD values, providing valuable insights into bone density and architectural integrity, and has shown sensitivity in detecting bone alterations in bruxism patients. Building on this robust methodological foundation, our study aimed to monitor trabecular bone changes following BTX-A treatment, reinforcing FA’s potential as a clinically useful tool in bruxism management.

The primary finding of our study indicates that administration of BTX-A resulted in measurable alterations in the structure of mandibular trabecular bone, as evidenced by statistically significant reductions in FD values in both the right and left angulus regions six months following treatment (*p* < 0.001). Specifically, the mean FD value on the right side decreased from 1.3856 to 1.2995, whereas on the left side, it declined from 1.3718 to 1.2529. These findings substantiate the hypothesis that diminished masseter muscle volume reduces mechanical loading on adjacent bone, thereby facilitating trabecular remodeling. This reduction was most pronounced in the angulus area, which can be attributed to the anatomical and functional significance of this region where the masseter muscle inserts and exerts substantial mechanical force during mastication. Since BTX-A temporarily inhibits muscle contraction, a marked reduction in biomechanical loading occurs at the angulus, potentially leading to decreased bone remodeling activity in this specific site. Previous biomechanical studies have confirmed that reduced mechanical stimulation, particularly in high-stress insertion zones, can lead to localized decreases in bone density and trabecular complexity [20]. The observed regional variability in FD change also emphasizes the importance of site-specific analysis in assessing skeletal responses to neuromuscular interventions, suggesting the angulus region may serve as a sensitive radiographic indicator of functional bone remodeling following masticatory muscle inactivation. This observation aligns with existing literature, wherein Lee et al. (2017) reported reductions in both muscle thickness and bone density subsequent to BTX-A injections [9]. Similarly, To et al. (2001) demonstrated a significant decrease in masseter muscle volume following BTX-A treatment, with effects remaining stable for 12 months, thus indicating a measurable impact on the underlying microarchitecture of bone [21].

When comparing the postoperative FD values between the group treated with BTX-A for bruxism and the control group without bruxism, no statistically significant differences were observed across any of the examined mandibular regions (condyle, ramus, angulus) (*p* > 0.05). This suggests that BTX-A treatment may assist in normalizing or maintaining the microarchitecture of mandibular trabecular bone in patients with bruxism, bringing it into alignment with that of individuals without the condition. These findings are consistent with those reported by Polat Balkan et al. (2024), who observed that the mean FD value of the BTX-A group (1.039) on second panoramic radiographs was comparable to that of the control group (1.033), indicating that BTX-A injections might contribute to sustaining normal bone density [11]. Consequently, these results further endorse the therapeutic potential of BTX-A in mitigating the adverse skeletal effects associated with bruxism.

Contrary to our findings, Polat Balkan et al. (2024) documented that the FD values for the angle and ramus in untreated bruxism patients were notably higher on the second panoramic radiograph compared to the initial one [11]. This disparity emphasizes the influence of persistent muscle hyperactivity on bone density. Moreover, their research indicated that the FD values in the angle of the BTX-A injection group were significantly reduced on the second panoramic radiograph (1.039) relative to the first (1.115). While our investigation observed a significant decrease solely in the angulus region following BTX-A administration, the overall trend of diminished FD values post-BTX-A across both studies suggests a consistent physiological response of bone tissue to decreased mechanical loading.

A further investigation by Unal Erzurumlu et al. (2023) indicated that FD in the gonial region was markedly lower in individuals with bruxism compared to those without, implying a more simplified internal configuration of trabecular bone among bruxers [22]. This finding contrasts with the results of Polat Balkan et al. (2024), who observed higher FD values in untreated bruxism cases, thereby underscoring the complex and occasionally conflicting nature of research findings regarding the influence of bruxism on bone microstructure [11]. In line with the observations of Polat Balkan et al. (2024) regarding the effects of intervention, our study demonstrated a significant reduction in fractal dimension in the angulus region following BTX-A administration. Furthermore, our finding that postoperative FD values in the BTX-A-treated bruxism group exhibited no statistically significant differences compared to the non-bruxism control group aligns with Polat Balkan et al.’s (2024) observation that BTX-A treatment can help maintain bone density within normal ranges [11]. Such discrepancies in findings on untreated bruxism, as well as the consistent effects of intervention, may be ascribed to variations in patient demographics, assessments of bruxism severity, or differences in fractal analysis methodologies—including region of interest selection and image processing protocols.

One of the most noteworthy aspects of our study is its ability to quantitatively evaluate the effects of BTX-A on mandibular bone microarchitecture through FA of panoramic radiographs. While the existing literature has primarily focused on BTX-A’s impact on muscle volume, facial aesthetics, or electromyographic activity, objective radiological assessments of skeletal effects remain scarce. Review articles, such as Balanta-Melo et al. (2019), emphasize that BTX-A-induced masticatory muscle atrophy promotes mandibular bone loss, affecting cellular and metabolic changes, microstructure degradation, and morphological alterations [23]. Experimental studies on animal models have demonstrated that BTX-A injections can significantly reduce mandibular ramus height and bigonial width in developing rats, and may cause 20–35% bone mass loss in the condylar and alveolar regions in adults. Furthermore, Tsai et al. (2011) observed reduced cortical bone thickness and bone mineral density (BMD) of the skull and mandibular bone structure in growing rats after BTX-A injection into masticatory muscles, confirming that masticatory hypofunction affects bone structure during development [24]. These results, especially the decrease in BMD around muscle attachment sites, strongly support our findings on FD reduction indicating bone changes. This highlights the crucial role of mechanical loading from muscles in maintaining bone health, even in non-weight-bearing bones like the mandible. In this context, our retrospective matched-pair design and region-specific FA allowed us to observe quantifiable changes in the trabecular structure of areas like the angulus. Since bone is a highly adaptive tissue, its microarchitecture responds dynamically to functional stimuli. Therefore, our findings suggest that the effects of BTX-A go beyond temporary muscular paralysis and have measurable impacts on bone remodeling, providing valuable insights into the muscle–bone functional relationship.

A systematic review conducted by Wojtovicz et al. (2024) revealed no significant alterations in most studies concerning mandibular condyle volume, density, and angle thickness following BTX-A application, often noting ambiguous clinical relevance and concluding “no clear pattern” for bone resorption [25]. However, the same review cited Lee et al. (2017), indicating greater mandibular angle volume reduction with repeated injections [9]. Balanta-Melo et al. (2019) affirmed consistent bone loss in animal models but highlighted limited and contrasting human study data [23]. Owen et al. (2022) supported that most evidence suggests BTX-A causes measurable, radiographically evident bone alterations (volume, cortical thickness, density) in regions like the condylar head and ramus, despite the low-to-very low quality of current studies due to inconsistencies [26]. Our findings, demonstrating a significant reduction in FD within the angulus region, contribute to the expanding corpus of human evidence suggesting that BTX-A indeed influences bone microarchitecture, potentially through reduced mechanical loading.

### 4.1. Limitations

This study possesses several limitations that warrant acknowledgment. Firstly, the retrospective design inherently constrains the capacity to control for confounding variables, such as bruxism severity, occlusal discrepancies, or parafunctional habits, which may influence the extent of mandibular bone remodeling. Despite our efforts to control for systemic diseases and medications through strict exclusion criteria, other factors such as patients’ dietary habits, physical activity levels, or smoking status could not be fully accounted for due to the retrospective nature of the data. Furthermore, while our within-group analysis showed high statistical power, the between-group comparison exhibited a power of only 60%. This low statistical power increases the risk of a Type II error, meaning that a true difference between the BTX-A and control groups might have gone undetected. Future prospective studies with larger and more balanced sample sizes are needed to confirm these findings and reduce the risk of false-negative results. Secondly, while panoramic radiography is a practical and extensively employed imaging modality, it offers two-dimensional representations that may not fully depict three-dimensional structural alterations within the mandible. Advanced imaging techniques such as cone-beam computed tomography (CBCT) or magnetic resonance imaging (MRI) could afford a more comprehensive assessment of volumetric and architectural bone modifications; however, these were not employed due to ethical and practical considerations in routine dental follow-up. Fourth, the patient cohort was predominantly female, thereby limiting the generalizability of the findings to the male population. Finally, the follow-up period, although adequate for observing short- to medium-term changes, may not encompass potential delayed or reversible alterations in bone quality. It is recommended that future prospective, controlled studies with extended observation durations, a more balanced sex distribution, and multimodal imaging techniques be conducted to validate and expand upon these findings. Such investigations could also examine more advanced diagnostic modalities such as dual-energy X-ray absorptiometry (DEXA) of the mandible or biochemical bone turnover markers (BTMs) to facilitate a more accurate assessment of bone density and remodeling, as suggested by Owen et al. (2022) [26].

### 4.2. Clinical Implications

The most significant finding of this research is the quantitative evaluation of BTX-A’s impact on mandibular bone microarchitecture through a novel application of fractal dimension analysis. This finding is of considerable clinical significance as it suggests that BTX-A treatment does not simply reduce muscle bulk but also has a measurable, and potentially predictable, effect on the underlying bone structure. Clinicians should be aware of this muscle–bone interaction when planning long-term BTX-A therapy.

While prior research has predominantly concentrated on BTX-A’s impact on muscle volume reduction, facial contouring, or electromyographic responses, our investigation provides a unique opportunity to examine the structural effects on bone tissue. By quantitatively assessing changes in FD values across designated mandibular regions, our study offers a radiographic proxy for skeletal adaptation resulting from neuromuscular modulation. This methodology not only supplements existing evidence on BTX-A-induced muscular atrophy but also introduces a novel, non-invasive approach for monitoring its potential skeletal side effects in dental practice.

Given the increasing popularity of BTX-A in both medical and aesthetic dentistry, incorporating FD analysis into post-treatment surveillance protocols could equip clinicians with a practical tool to safeguard long-term osseous health. Ultimately, this study’s findings provide a strong rationale for integrating objective bone analysis into routine bruxism management, enabling a more holistic and evidence-based approach to patient care.

## 5. Conclusions

This study provides compelling evidence that BTX-A administration leads to significant, region-specific reductions in mandibular trabecular bone fractal dimension, particularly in the angulus region. Our findings demonstrate that BTX-A treatment in bruxism patients results in a trabecular bone microarchitecture that is quantitatively similar to that of non-bruxism individuals. This suggests that the therapy may play a role in normalizing bone structure by mitigating the excessive mechanical load associated with bruxism. By employing fractal dimension analysis on panoramic radiographs, our research offers a novel and non-invasive method for the objective evaluation of bone remodeling in response to neuromuscular interventions. This approach provides a radiographic proxy for skeletal adaptation and highlights the clinical relevance of integrating quantitative, image-based evaluation methods into post-treatment follow-up. In conclusion, this study underscores the importance of considering the skeletal effects of BTX-A treatment. Incorporating quantitative and non-invasive tools like FD analysis into routine practice may facilitate the early detection of osseous adaptations and enhance the precision of treatment planning in dental and maxillofacial disciplines.

## Figures and Tables

**Table 1:** Changes in mandibular fractal dimensions following botox administration in bruxism cases.

Region	Pre-op (Mean ± SD)	Post-op (Mean ± SD)	*p*
Right Condyle	1.2906 ± 0.1078	1.2991 ± 0.1084	0.665
Right Ramus	1.3255 ± 0.0996	1.3121 ± 0.1267	0.558
Right Angle	1.3856 ± 0.0896	1.2995 ± 0.1225	0.000 *
Left Condyle	1.2876 ± 0.1008	1.3033 ± 0.1086	0.423
Left Ramus	1.3538 ± 0.0968	1.3137 ± 0.1002	0.063
Left Angle	1.3718 ± 0.0726	1.2529 ± 0.1024	0.000 *

* *p* < 0.05.

**Table 2:** Comparative assessment of mandibular postoperative FD values between bruxism patients receiving botulinum toxin therapy and non-bruxism controls.

Region	Botox Group (Mean ± SD)	Control Group (Mean ± SD)	*p*
Right Condyle	1.2991 ± 0.1084	1.3009 ± 0.0418	0.932
Right Ramus	1.3121 ± 0.1267	1.3031 ± 0.0690	0.716
Right Angle	1.2995 ± 0.1225	1.3312 ± 0.0464	0.168
Left Condyle	1.3033 ± 0.1086	1.2896 ± 0.0416	0.503
Left Ramus	1.3137 ± 0.1002	1.3225 ± 0.0691	0.673
Left Angle	1.2529 ± 0.1024	1.2513 ± 0.0464	0.934

## Data Availability

The data presented in this study are not publicly available due to ethical and privacy restrictions. However, anonymized datasets may be made available from the corresponding author upon reasonable request and with appropriate institutional approval.

## Abbreviations

AAOP	American Academy of Orofacial Pain
BMD	Bone Mineral Density
BTX-A	Botulinum Toxin Type A
DEXA	Dual-Energy X-ray Absorptiometry
FA	Fractal Analysis
FD	Fractal Dimension
HU	Hounsfield Units
ICC	Intraclass Correlation Coefficient
MRI	Magnetic Resonance Imaging
PRs	Panoramic Radiographs
ROIs	Regions of Interest
SD	Standard Deviation
TMJ	Temporomandibular Joint
